# Ancient Duplication and Lineage-Specific Transposition Determine Evolutionary Trajectory of *ERF* Subfamily across Angiosperms

**DOI:** 10.3390/ijms25073941

**Published:** 2024-04-01

**Authors:** Xun-Ge Zhu, Ge-Ran Hutang, Li-Zhi Gao

**Affiliations:** 1 Germplasm Bank of Wild Species in Southwestern China, Kunming Institute of Botany, The Chinese Academy of Sciences, Kunming 650201, China; zhuxunge@mail.kib.ac.cn; 2 University of Chinese Academy of Sciences, Beijing 100049, China; 3 Institute of Forest Industry, Yunnan Academy of Forestry and Grassland Science, Kunming 650201, China; hutanggeran@yafg.ac.cn; 4 Engineering Research Center for Selecting and Breeding New Tropical Crop Varieties, Ministry of Education, Tropical Biodiversity and Genomics Research Center, Hainan University, Haikou 570228, China

**Keywords:** *ERF* subfamily, gene duplication, synteny network, evolution, angiosperm

## Abstract

*AP2*/*ERF* transcription factor family plays an important role in plant development and stress responses. Previous studies have shed light on the evolutionary trajectory of the *AP2* and *DREB* subfamilies. However, knowledge about the evolutionary history of the *ERF* subfamily in angiosperms still remains limited. In this study, we performed a comprehensive analysis of the *ERF* subfamily from 107 representative angiosperm species by combining phylogenomic and synteny network approaches. We observed that the expansion of the *ERF* subfamily was driven not only by whole-genome duplication (WGD) but also by tandem duplication (TD) and transposition duplication events. We also found multiple transposition events in Poaceae, Brassicaceae, Poales, Brassicales, and Commelinids. These events may have had notable impacts on copy number variation and subsequent functional divergence of the *ERF* subfamily. Moreover, we observed a number of ancient tandem duplications occurred in the *ERF* subfamily across angiosperms, e.g., in Subgroup IX, IXb originated from ancient tandem duplication events within IXa. These findings together provide novel insights into the evolution of this important transcription factor family.

## 1. Introduction

Due to their sessile nature, plants must directly face environmental insults such as low temperature, drought, and high salt, which can impact their growth, expansion and yield [1]. To survive and reproduce at various ecological niches, plants have evolved multiple adaptive traits and complex signaling and regulatory systems, which are often regulated by transcription factors [2].

The *APETALA2*/*ETHYLENE-RESPONSIVE FACTOR* (*AP2*/*ERF*) superfamily is one of the most prominent transcription factor families regulating plant development and stress responses. It has been hypothesized that the *AP2*/*ERF* family of transcription factors originated from the horizontal transfer of bacterial or virus HNH-AP2 endonuclease via transposition and the homing process [3].

According to the number of AP2 domains, it can be divided into three main subfamilies: the *AP2*, *RAV,* and *ERF* subfamilies [4]. The *AP2* subfamily comprises proteins with two highly similar and serially repeated AP2 domains. Most of the known functions of *AP2*-like genes are important for developmental processes [5]. The *RAV* subfamily contains one AP2 domain and one B3 domain. The members of the *RAV* subfamily play important roles in plant physiological processes, such as leaf senescence, flowering development, organ growth, and hormone signaling [6,7]. The *ERF* subfamily comprises one AP2 domain, and numerous studies indicate their involvement in various biotic and abiotic stresses in plants. According to the difference in the 14th and 19th amino acid residues in the AP2 domain, *ERF* is divided into *DREB* and *ERF* subfamilies [8]. Based on exon/intron and protein motif structural analyses. Nakano et al. [4] further classified *ERF* into ten groups (I–X), with I–IV as the *DREB* subfamily and V–X as the *ERF* subfamily. Note that the *ERF* mentioned below is a term for the *ERF* subfamily. Many studies have shown that lots of genes of the *DREB* subfamily are involved in plant stress response [9]. For example, *DREB1*/*CBF* proteins belonging to the *DREB* family have been identified as key transcription factors involved in cold acclimation [10,11]. *DREB2*-type gene expression was induced by salt, heat, and dehydration [12,13]. *ERF* also plays an important role in plant response to biotic and abiotic stresses. *ERF*-VII transcription factor family was involved in adaptation to flooding and its associated stresses [14]. Among them, *ERF105* plays an important role in *Arabidopsis* freezing tolerance and cold acclimation [15]. *RAP2.6* plays a key role in responding to ABA, JA, wounding, cold, drought, and salinity stresses [16]. *SHINE* and its rice homolog *OsWR1* enhance the tolerance of plants to drought by influencing wax synthesis [17,18]. *ZmERF1* is activated in response to drought stress via the ethylene and ABA signaling pathways [19]. At the same time, some functions of the *ERF* genes are closely related to plant growth and development. For example, *PUCHI* regulates lateral root development, floral meristem identity, and organ initiation in *Arabidopsis thaliana* [20,21]. *ESR1* induces the initiation of shoot regeneration [22,23]. The subgroup *ERF*-VIIIa in *A. thaliana*, rice, and tobacco had a cell death-inducing ability [24].

While more and more high-quality reference genomes were sequenced, *AP2*/*ERF* genes were identified in more plant species, providing unprecedented opportunities to study the evolution of *AP2*/*ERF* in flowering plants. Some studies have explored the evolutionary history of the *AP2* and *DREB* families in plants [25,26,27], while others focused on a specific lineage or subclade of *AP2*/*ERF* genes [24,28,29]. However, the evolutionary history of the *ERF* subfamily has not been comprehensively investigated in angiosperms.

Gene duplication, including whole-genome duplication (WGD), segmental duplication (SD), tandem duplication (TD), and transposition duplication [30,31,32], is considered to be a driving force in gene family evolution, providing raw genetic materials for the evolution of functional novelty [33,34,35]. Previous studies have shown that lineage-specific WGD plays an important role in the amplification of *AP2*/*ERF* genes [36]. Tandem duplication also has important effects on the amplification of Subgroups III and IX of *AP2*/*ERF* [37]. However, the influence of transposition duplication on *AP2*/*ERF* genes, for example, has not been examined.

Genomic synteny information holds significant value in comparative genomics analysis, enabling the examination of both large-scale (e.g., genome rearrangement and duplication events) and small-scale (e.g., gene transpositions, insertions, and deletions) molecular evolution events among species [38]. Previous approaches to genomic synteny analysis of target gene families focused on a small number of species, showing pairwise syntenic relationships. With the increase of genomes that are simultaneously analyzed, it becomes increasingly more difficult to organize and display such syntenic relationships. The Synteny networks method [39] overcomes challenges imposed by pairwise interspecies comparisons, enabling the identification of previously undiscovered and complex evolutionary relationships in various gene families. This approach is particularly instrumental in unveiling pivotal events related to gene duplication and transposition [40]. Its efficacy has been demonstrated in studies of multiple gene families [40,41,42].

In spite of understanding the *AP2*/*ERF* evolution to some extent, there is still a lack of knowledge about the evolutionary trajectory of the *ERF* subfamily. Besides WGDs, are there any other types of gene duplications affect the number of *ERF* family members? How is lineage-specific expansion achieved, and how does it impact gene functional divergence? How did the genes of each *ERF* subgroup originate? Answering these questions often requires a wealth of genomic data. Leveraging on the increasing public release of high-quality genomes representing the major lineages of angiosperms, in this study, we aim to address these questions using 107 high-quality genomes on behalf of the major lineages of angiosperms in conjunction with phylogenomic and synteny network approaches.

## 2. Results

### 2.1. ERF Gene Copy Number Variation in Angiosperms

**Figure 1:**
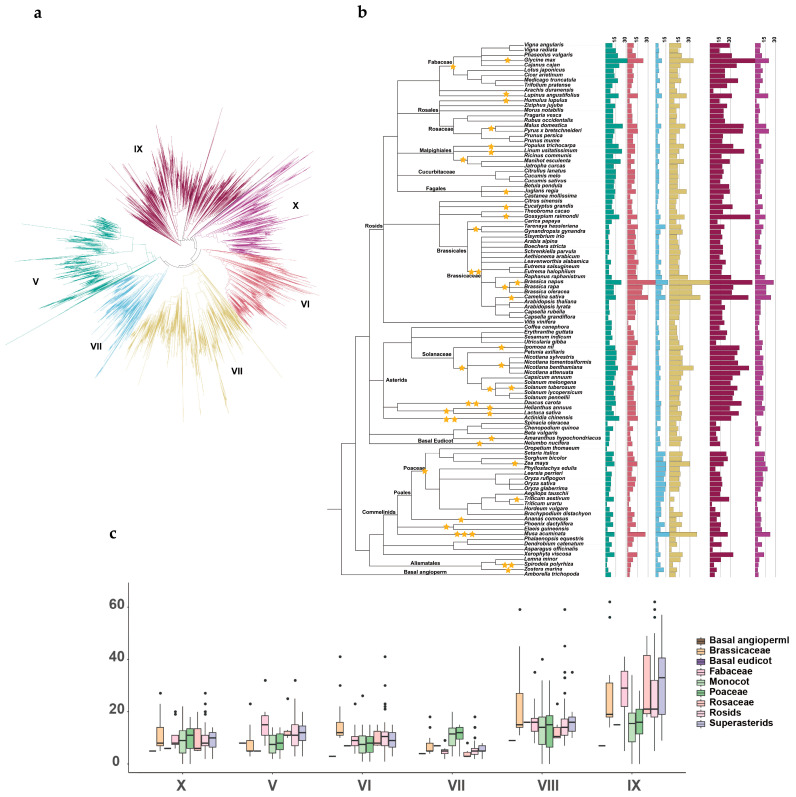
Phylogenomic analysis and copy number distribution of the *ERF* subfamily across angiosperms. (**a**) Maximum-likelihood gene tree illustrating the relationships within the *ERF* gene family. The six subgroups are represented by different colors. (**b**) Bar chart displaying the copy numbers of Subgroups V–X across different plant species. Yellow pentagrams represent whole-genome duplication events. (**c**) Comparison of *ERF* gene numbers among the six subgroups across different lineages.

To explore the evolutionary history of the *ERF* subfamily in angiosperms, a total of 8163 *ERF* genes were identified from 107 angiosperm species (Tables S1–S3). A phylogenetic tree was constructed using all protein sequences. Based on the classification of *ERF* genes in *A. thaliana* and rice [4], they were divided into six subgroups, V–X (Figure 1a). The number of *ERF* genes varied greatly across angiosperms (Figure 1b). *Oropetium thomaeum* had only six *ERF* genes, of which none were identified in Subgroups VIII, IX, and X. *Brassica napus* had the highest number of *ERF* genes, totaling 230 genes. The distribution of *ERF* genes varied unevenly among the subgroups, with the highest number in Subgroup IX, followed by Subgroups VIII, V, VI, X, and VII. Additionally, we found that several plant species with large numbers of *ERF* genes were associated with the occurrence of recent WGD events. There were, for example, 185, 119, 183, 122, 127, 230, 107, and 147 genes identified in *Glycine max*, *Lupinus angustifolius*, *Camelina sativa*, *Brassica oleracea*, *Brassica rapa*, *Brassica napus*, *Zea mays*, and *Musa acuminata*, respectively (Figure 1b; Table S2).

Furthermore, we investigated the variation in *ERF* gene copy number among the six subgroups across these lineages. Our results indicated remarkable differences in copy numbers from one lineage to another. The comparison between monocots and eudicots showed that the overall number of *ERF* genes was higher in eudicots (31 to 230) than in monocots (6 to 147). However, Subgroup VII exhibited a contrasting trend, with a larger number of genes in monocots (3 to 20) than in eudicots (2 to 18). The result suggested that Subgroup VII in monocots retained a large number of genes as a result of independent duplication events after the split of monocots and eudicots. Additionally, within Subgroup V, legume plants had a large number of genes, ranging from 7 to 32. In Subgroups VI and VIII, *Brassicaceae* species had a larger number of genes, ranging from 10 to 41 and 11 to 45, respectively (Figure 1b,c; Table S2).

### 2.2. Conservation and Dynamics of ERF Genes

**Figure 2:**
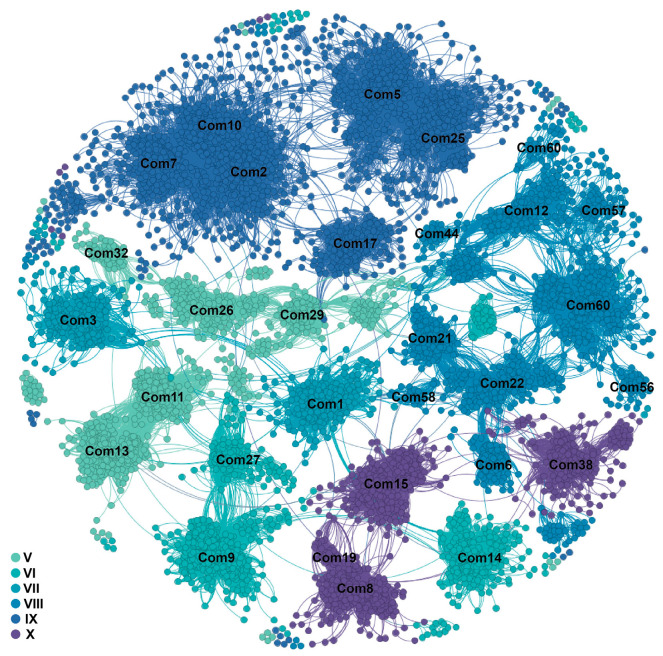
Synteny network graph illustrating genomic collinearity relationships between genes. Nodes represent genes, and edges represent collinearity relationships. The six subgroups are depicted using different colors. Com is denoted for the community.

**Figure 3:**
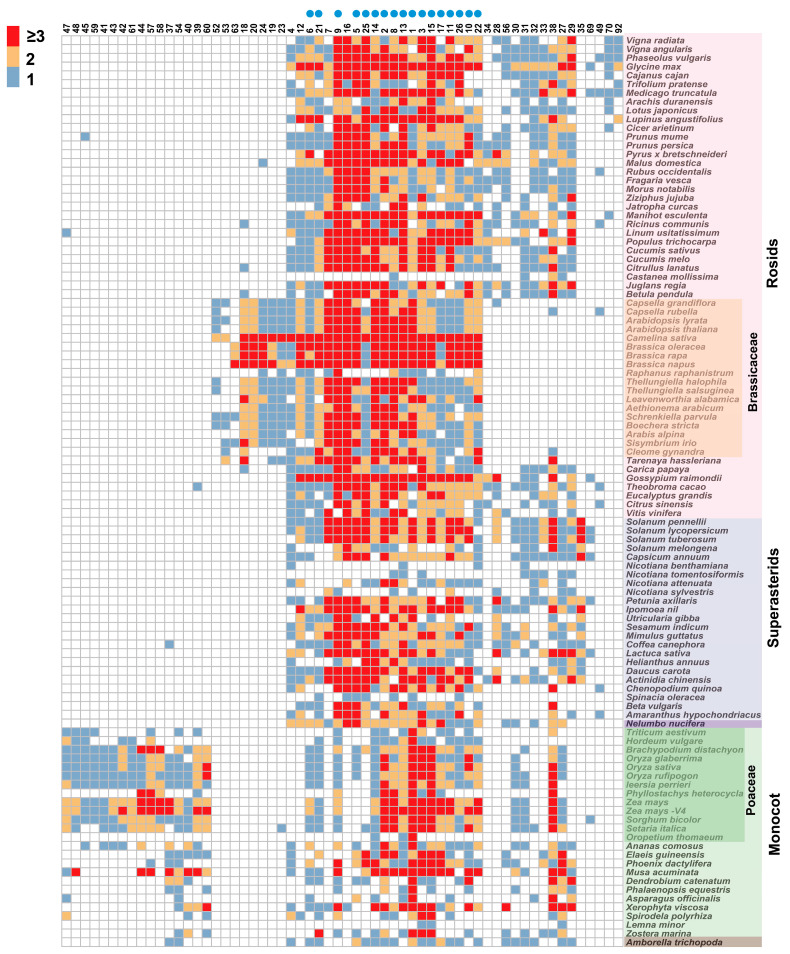
Phylogenetic profiling showing the number and distribution of syntenic *ERF* genes in angiosperms. Rows represent species, and columns indicate species. synteny communities species. The numerical labels at the top correspond to the numerical labels of the communities. Only communities with gene numbers greater than or equal to nine are displayed. The blue circles at the top of the diagram represent that there is at least one gene copy in the represented lineage.

To search the conservation and dynamics of *ERF* genes in a genomic context across angiosperms, we constructed a synteny network using data from 107 plant genomes (Figure 2; Table S4). Our results revealed weak syntenic connections among the six subgroups. Syntenic connections were primarily observed within the same subclades within each subgroup and, to some extent, between different subclades (Figure 1a and Figure 2). Subsequently, we used phylogenetic profiling to visualize the conservation and dynamics of *ERF* genomic contexts (Figure 3; Table S5). Here, each column represents a syntenic occurrence for a certain *ERF* gene community in each plant species. We categorized the community into two major types: angiosperm-wide synteny and lineage-specific synteny. Angiosperm-wide synteny indicated the presence of genes across the entire angio-sperm lineagereferred to syntenic relationships existing between one or more monocots and eudicots, reflecting ancient conservation in genomic contexts, including communities 1, 2, 3, 5, 8, 9, 11, 13, and so on. Lineage-specific synteny, on the other hand, suggested synteny relationships specific to certain lineages, highlighting the dynamics of genomic contexts. Communities 20 and 24 were Brassicaceae-specific; communities 59 and 61 were Poaceae-specific; communities 41, 42, and 43 were Polea-specific; communities 39, 40, and 60 were monocot-specific; and community 35 was asterids-specific. The observed lineage-specific synteny suggested that gene duplication events other than whole genome duplications have significantly contributed to the expansion of the ERF subfamily in angiosperms.

### 2.3. Phylogenomic Synteny Network Analyses of ERF Genes

**Figure 4:**
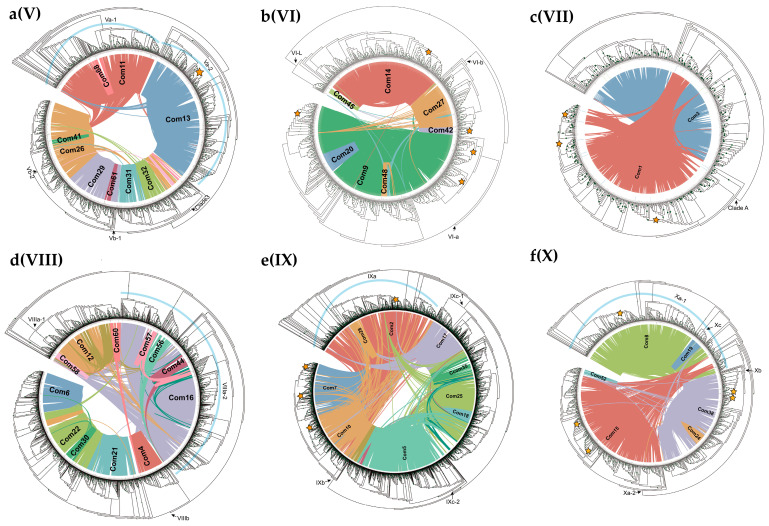
Phylogenomic and synteny network analyses for each of the six *ERF* subgroups in angiosperms. (**a**–**f**) Maximum-likelihood trees for each of the six *ERF* subgroups. Genes detected in syntenic genomic blocks are connected using curved lines. The syntenic connections belonging to different synteny network communities are plotted using different colors. Synteny network communities are numbered according to those depicted in Figure 3. Putative WGD events that occurred in Brassicaceae and Poaceae are indicated by pentagrams. Com is denoted for the community.

To further understand the evolutionary history of subgroups, we explored each subgroup using phylogenomic synteny network analyses. We divided Subgroup V into four clades by integrating the synteny network and phylogenetic profiling analyses (Figure 3 and Figure 4a). Clade 1, referred to as Va-1, encompassed a subset of Va members associated with communities 11 and 54; community 11 was angiosperm-wide. Clade 2 corresponded to the remaining Va members; we here renamed it Va-2 corresponding to community 13. They formed a highly supported monophyletic clade. Community 13 was angiosperm-wide. The result showed that the Va members contained two ancestral types. Clade 3 formed a highly supported clade corresponding to communities 31, 32, and 34; No members of the Brassicaceae family were found in this clade. community 32 was rarely found in monocots and did not contain any member genes of Brassicaceae. Clade 4 corresponded to Vb, which was further divided into two subclades, Vb-1 and Vb-2. Vb-1 correlated with communities 29 and 61. Community 29 did not contain any genes from Brassicaceae and Poaceae. Within this clade, however, genes from Poaceae formed a highly credible clade; community 61 was mapped to this clade. The results showed that transposition events occurred in the ancestors of Poaceae. Vb-2 contained communities 26 and 41. Community 26 was angiosperm-wide. Community 41 was Poales-specific and formed a highly confident clade, suggesting a transposition event occurred in the ancestor of Poales.

Subgroup VI, also known as the *CRF* family, can be divided into two clades (Figure 4b). Clade 1 corresponded to Subgroup VI, while Clade 2 corresponded to Subgroup VI–L. Clade 1 further branched into VIa and VIb. VIa included communities 9, 20, and 48. Community 9 was the largest community in this subgroup, consisting of 420 nodes. Brassicaceae-specific WGD events were observed, and *AT3G61630*/*AT2G46310*, *AT4G27950*/*AT5G53290*, and *AT4G23750*/*AT4G11140* were retained after experiencing α/β WGD events, but no Poaceae-specific WGD was observed (Figure 4b). The result suggested that members of the VIa exhibited dissimilar expansion mecha-nisms in Brassicaceae and Poaceae. VIb was associated with communities 27 and 42. No Brassicaceae gene was found in VIb, suggesting a rapid loss of VIb genes during evolution in Brassicaceae. A transposition event occurred in the Poales was observed, and these genes were re-tained after the specific WGD event in Poaceae (Figure 3 and Figure 4b). 

Subgroup VII can be divided into the two major clades, Clade A and Clade B, primarily corresponding to communities 1 and 3, respectively (Figure 3 and Figure 4c). These two clades encompassed 79% of genes in Subgroup VII, indicating strong conservation compared to other subgroups. Interestingly, our analysis revealed that monocots had significantly larger numbers of VII subgroups compared to eudicots. In Clade A, the number of genes in rice was three times more than that in *A. thaliana*. However, we failed to observe such a result in Clade B. These results suggested that the expansion occurred mainly in Clade A, which might have played an important role in the adaptive evolution of Poaceae. In addition, both community 1 and community 3 clades contained basal angiosperm, indicating that they might originate from two distinct ancestral types.

Combining the results derived from phylogenomic and synteny network analyses, three clades could be identified (Figure 3 and Figure 4d). Clade 1 formed a monophyletic clade containing two VIIIa members, named VIIIa-1, corresponding to communities 12, 58, and 60. They all were lineage-specific communities. Community 16 was the largest and most significant one, with 388 nodes, which primarily included eudicots. This result suggested that VIIIa-2 underwent specific expansion in eudicots. Clade 3 corresponded to VIIIb and contained multiple angiosperm-wide communities. These results together suggested that rapid gains and losses have occurred along different lineages, making the evolutionary history of Subgroup VIII become fairly confusing and complex.

IX is the largest subgroup within the *ERF* subfamily. Nakano et al. [4] divided it into three subgroups (IXa, IXb, and IXc). By combining phylogenomic and synteny network analyses, we divided IX into four clades (Figure 3 and Figure 4e). Clade 1, corresponding to IXa, mainly contained members of communities 2 and 28. Community 2 was angiosperm-wide, and specific WGD was observed in Brassicaceae (Figure 4e). Clade 2 contained communities 17, 18, 25, and 35, representing Subgroup IXc, which was renamed IXc-1. Communities 17 and 25 were angiosperm-wide. We also observed two transposition events occurred separately in Brassicales and the asterids. Clade 3 only contained community 5 and represented additional IXc members, which was renamed IXc-2 in this study. These results demonstrated the conservation of IXc members in angiosperms, indicating that they had two ancient ancestral types. Clade 4 corresponded to IXb, and communities 7 and 10 were mapped to this clade; community 10 was angiosperm-wide; community 7 was eudicot-specific.

According to the results of the phylogenomic and synteny network analyses, Subgroup X could be divided into three clades (Figure 3 and Figure 4f). Clade 1 was associated with communities 8 and 19. Community 8 was angiosperm-wide, including some Xa members, which was renamed Xa-1. Community 19 corresponded to Xc and was Brassicaceae-specific, suggesting that Xc was unique to Brassicaceae and originated from the transposition event in the ancestors of Brassicaceae. Clade 2 corresponded to Xb and contained communities 38 and 24. Most members of Xb were found in community 38, while members of Brassicaceae formed a distinct community (community 24), suggesting that Xb had transposed in the ancestors of Brassicaceae, resulting in a change in position. Clade 3 corresponded to the remaining Xa members, which was named Xa-2; communities 15 and 52 were mapped to this clade. Community 15 was angiosperm-wide. Community 52 was Brassicaceae-specific. These results showed that there were two ancestral types of the Xa subgroup.

### 2.4. Ancient Tandem Duplications

**Figure 5:**
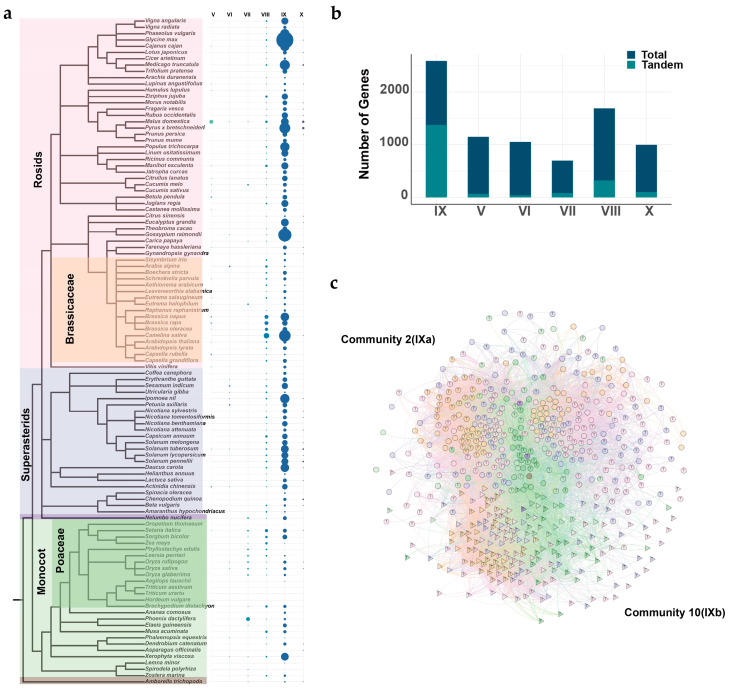
Identification of tandem duplicated genes. (**a**) Distribution of tandem duplicated genes among the six subgroups, with the size of the circles representing the number of tandem duplicated gene pairs. (**b**) Proportions of tandem duplicated genes in each of the six subgroups. (**c**) Close-up view of the community that generated tandem duplication events, where node shapes represent different communities, node colors represent different lineages, and nodes labeled with ‘T’ represent tandem duplication genes.

We investigated tandem duplications that occurred in the *ERF* subfamily. Our findings revealed that tandem duplications made, to variable extents, contributions to the expansion of different subgroups. Subgroup IX exhibited a substantial number of tandem duplicated genes (Figure 5a; Table S6), which is consistent with the previous study [37]. Such tandem duplicated genes accounted for approximately 53% of Subgroup IX, while Subgroup VIII had 19% of tandem duplicated gene pairs (Figure 5b; Table S6). Notably, tandem duplicated genes in Subgroup VII were predominantly enriched in Poaceae (Figure 5a), indicating that tandem gene duplication acted as a driving force of the amplification of Subgroup VII in Poaceae. In Subgroup V, we interestingly observed an ancient tandem duplication event in the ancestor of Brassicaceae (Figure S1a; Table S7). Similarly, an ancient tandem duplication event occurred separately in the ancestors of monocots and eudicots in VIIIa-2 (Figure S1b; Table S7). Surprisingly, we identified an ancient tandem duplication event involved in IXa and IXb (Figure 5c; Table S7), suggesting that IXb may have originated from this tandem duplication event. Our further analysis, however, failed to detect it in basal angiosperms. Instead, we found gene pairs derived from tandem duplications between IXa and IXb in both early eudicots and monocots. The observations suggested that the tandem duplication event occurred after the divergence of basal angiosperms and the ancestors of monocots and eudicots.

## 3. Discussion

Whole-genome duplications in plants were found to occur non-randomly, primarily during three periods (~120 million years ago [Ma], ~66 Ma, and <20 Ma), suggesting essential roles of WGDs under environmental selection [43]. Three rounds of WGD events were identified in Brassicaceae (γ-β-α) and Poaceae (τ-σ-ρ) [44,45], while recent WGD events were detected in many plant species [46]. During subsequent fractionation and diploidization, gene loss was the most common evolutionary fate, with a large proportion of genes returning to a single-copy state [47,48]. The retained duplicated genes are considered particularly important for functional innovation via neo-functionalization or sub-functionalization [48]. In this study, we observed the retention of *ERF* genes after σ and ρ WGD events. Our results also showed that plant species that underwent recent WGD events exhibited a higher number of *ERF* genes compared to other species. Previous studies reported that transcription factors are among these genes that are retained to a greater extent following WGD events [30,49].

Previous studies suggested that the expansion of the *ERF* genes has made great contributions to plant environmental adaptation [36,50,51]. In this study, we compared copy number variation among the six *ERF* subgroups across angiosperms. Except for VII, we found that the number of genes in eudicots was higher than that in monocots. Our further analysis showed that the VIIa genes retained a large number of copies after Poaceae experienced τ-σ-ρ WGDs, TD, and transposition. In rice, VII genes were reported to be involved in water (flood and drought) and salt stresses [52,53,54], suggesting the occurrence of functional differentiation. It is likely that the expansion of VIIa genes has played a pivotal role in adapting to dry and saline habitats in Poaceae.

The *ERF* subfamily was formerly classified into six subgroups, the V–X or B1–B6 [4,8]. Due to a limited number of genomes with relatively poor quality, such efforts lacked sufficient resolution of their evolutionary processes for each subgroup, ignoring the real evolutionary history of the gene family. By combining phylogenetic and synteny network analyses, however, evolutionary relationships of this gene family can be better reconstructed [39]. In this study, we examined the evolution of each subgroup by combining phylogenetic and synteny networks and revealed some unobserved evolutionary scenarios within the subgroups. For example, our work further divided Va into Va-1 and Va-2, which correspond to two different angiosperm-wide communities, indicating the existence of two distinct ancestral types. In Va-2 (community 13), three *A. thaliana* genes, namely *SHN1*, *SHN2*, and *SHN3*, were identified, which were previously classified as the *SHN* branch [17]. These genes possess conserved motifs in their central and C-terminal regions, which are associated with wax accumulation in leaves, enhancing drought tolerance in plants. Additional members of Va were distributed in Va-1 (community 11), which only contains a partial mm domain and cm domain. Previous studies showed the collinearity within VII among 16 angiosperms, which was divided into two synteny blocks, indicating that Subgroup VII of angiosperms originated from two ancestral genes [14]. It is further supported by our findings that Subgroup VII corresponded to two conserved communities. These two examples strongly support the rationale and accuracy of the classification in this study. Similar results were also observed in IXc and Xa, which were further divided into IXc-1, IXc-2, and Xa-1, Xa-2, respectively.

It is possible that they may have originated from WGD events in the ancestors of angiosperms. The *ERF* subfamilies were observed in major lineages of Viridiplantae, suggesting that they may have already formed different ancient archetypal genes before the divergence of angiosperm ancestors via a number of gene duplication and loss events. For example, VIIIb contained multiple angiosperm-wide communities, indicating that multiple ancient archetypal gene types might exist, indicating that ancient VIIIb genes may have undergone one or more gene duplication events before the divergence of angiosperms, accompanied by gene duplication and subsequent loss, making the evolutionary history of VIII more complicated as observed.

Transposition duplication is one of the most important modes of gene duplication that has often been overlooked in previous studies [36]. The genes of *ERF* subfamilies probably had only one intron or even none [28]. Retrotransposition acts as a major mechanism of gene duplication that results in intronless genes [55]. Thus, it is likely that some of the single AP2 domain genes were duplicated via retrotransposition. In this study, we demonstrated that transposition events have a significant impact on copy number variation. The Vb-1 genes transposed to another genomic context in the common ancestor of Poaceae. The ancestor of Brassicaceae might have also undergone the transposition from Vb-1 but was rapidly lost during evolution. In Vb-2, replicative transposition occurring in the common ancestor of Poales increased the copy number of this lineage. Transposition duplications were observed for VIa in both Brassicaceae and Poaceae. It is noteworthy that the evolutionary mechanisms of VIa differ between monocots and eudicots, with significant expansion of copy numbers in Brassicaceae being influenced by WGD, while in Poaceae, the changes in VIa were primarily caused by replicative transposition. VIb did not contain genes from Brassicaceae, whereas, in Poaceae, the genes in VIb expanded via replicative transposition and subsequent WGD events. We found transposition duplication occurred in Poales for VI–L. VIIIa did not exhibit conserved collinearity across all angiosperms but showed eudicot-specific synteny, indicating a more unstable genomic context for VIIIa. We also observed a transposition event in IXb that occurred in the ancestor of eudicots, and in *Arabidopsis thaliana*, we found that four gene copies were retained via an α/β WGD event. Functional experiments demonstrated that *ERF105* functioned in cold stress response, and its overexpression considerably enhanced freezing tolerance [15]. It is likely that the transposition event may enhance the adaptation of eudicots during global cooling, but functional experiments are needed to verify it in more species. In addition to a Brassicales-specific transposition in IXc-1, we also observed an asterids-specific transposition. Xc was a unique grouping found only in Brassicaceae, possibly originating from a transposition event in Xa. Changes in the genomic context of Brassicaceae occurred in Xb. In Xa-2, we observed a Brassicaceae-specific transposition. These results indicated that extensive transposition occurred in the ancestors of Brassicaceae/Brassicales and Poaceae/Poales. Other studies have also frequently observed active transposition events in Brassicaceae and Poaceae, and this pattern holds even when the number of genomes increased from 47 to 107 [40,41,56,57]. However, the mechanism by which transpositions are active in these two lineages remains unclear. In addition, based on the number of lineage-specific communities, we found transposition events that occurred in monocots appeared more active than in eudicots.

Tandem duplications are also an important mechanism leading to the expansion of gene families that respond to stress responses [58,59,60]. Previous studies have observed the enrichment of tandem gene duplications in Subgroup IX [37,61]. In this study, we identified a large number of tandem gene duplications, particularly in Subgroup IX, where tandem gene duplications accounted for over half of the subgroup. We found that Subgroup IXb originated from an ancient tandem duplication event involved in Subgroup IXa, followed by subsequent polyploidization. This differentiation likely occurred after the divergence of basal angiosperms and before the differentiation of monocots and eudicots. A similar mechanism was observed in *DREB*-III, where the *DREB*-IIIc genes originated from an ancient tandem duplication event in the ancestors of angiosperms and further evolved into innovations with cold-sensitive response, expanding stepwise in eudicots and monocots via independent duplications [62]. IXb is induced by various biotic and abiotic stresses [63,64], but whether it enhances adaptability during the radiation and diversification of angiosperms requires further functional experimental validation.

## 4. Materials and Methods

### 4.1. Plant Genomes Downloaded

A total of 107 genomes, including basal angiosperms, monocots, early eudicots, asterids, and rosids, were obtained from public repositories following the described method [65]. Protein sequences and GFF/GFF3 annotation files from fully sequenced genomes were downloaded. More detailed genomic information is presented in Table S1.

### 4.2. Identification of ERF Genes in 107 Plant Species

The HMMER (profile hidden Markov models) tool (v3.1) [66] was employed using AP2 (PF00847) downloaded from Pfam [67] to identify *AP2* homologous genes from each of the whole-genome datasets. Protein sequences containing two AP2 domains were classified as *AP2* subfamily members, and those with a single AP2 domain were classified as *ERF* subfamily members. All proteins with significant hits (e-value < 10^−5^) were used in this analysis. *ERF* protein sequences were extracted according to *ERF*-conserved amino acids.

### 4.3. Multiple Sequence Alignment and Phylogenetic Analysis

Full-length protein sequences were aligned using MAFFT with an auto-progressive algorithm with a gap penalty of 1.0 [68]. Gapped positions in the multiple sequence alignments were removed using trimAl [69], adjusting the ‘-gt’ and ‘-cons’ parameters to obtain reliable alignment results. Maximum-likelihood trees were constructed from trimmed alignments with IQ-TREE2 [70]. The optimal protein model was determined using the -MFP parameter, and 1000 ultrafast (-alrt) and 1000 bootstraps (-B) were performed. Phylogenetic trees were edited in ITOL 4.4 [71].

### 4.4. Genomic Synteny Network Construction

The synteny network method [39] was employed for syntenic block identification, network construction, and community detection. The SynNet-Pipeline (available at https://github.com/zhaotao1987/SynNet-Pipeline) (accessed on 11 March 2024) was used for this purpose. Initially, pairwise comparisons were performed using diamond [72]. Genomic collinearity between all pairwise genome combinations was computed using MCScanX [73] with default parameters (minimum match size for a collinear block = 5 genes; max gaps allowed = 25 genes). Syntenic blocks containing the identified *ERF* sequences were used to construct synteny networks, which were visualized and edited using Gephi 0.9.1 (https://gephi.org/) (accessed on 6 June 2023). An infomap implemented under the ‘igraph’ package in R 4.3.1 was employed to detect communities within the synteny networks. The synteny communities were further analyzed using phylogenetic profiling. This R script can be used to analyze cluster composition, calculate distance, and perform hierarchical clustering. All synteny communities were analyzed to determine the number of involved syntenic gene copies for each genome.

## 5. Conclusions

In this study, we comprehensively identified *ERF* genes in 107 plant genomes and elucidated the copy number variation within the *ERF* family across angiosperms. Comparative analyses among lineages revealed a contrasting trend in the copy number variation in the Subgroup VII genes compared to other subgroups, with higher gene numbers observed in monocots than in eudicots. Genomic synteny network analyses highlighted significant conservation among multiple subclades across angiosperms. Nevertheless, several subclades exhibited dynamic and distinct lineage-specific patterns. The combined analyses of phylogenomic and synteny networks unveiled the impact of ancient duplication and lineage-specific transposition on the expansion of *ERF* genes. Ancestral lineage-specific transpositions of *ERF* genes were identified in Poaceae, Brassicaceae, Poales, Brassicales, and Commelinids, contributing to the functional divergence among gene members of the family. The ancient tandem duplication events occurred during the evolution of Subgroup IX, with IXb arising from tandem duplication events in IXa. With the increasing release of high-quality genomes of non-angiosperm plants, future research endeavors will leverage the framework to further enhance the understanding of evolutionary patterns of *ERF* across the entire plant kingdom.

## Data Availability

The data presented in this study are available in the Supplementary Materials.

## Supplementary Materials

The following supporting information can be downloaded at https://www.mdpi.com/article/10.3390/ijms25073941/s1.
