# Supplementary material for: Ancient Duplication and Lineage-Specific Transposition Determine Evolutionary Trajectory of ERF Subfamily across Angiosperms
Source: Int J Mol Sci. 2024 Apr 1;25(7):3941. doi: 10.3390/ijms25073941 (PMC11011629; doi:10.3390/ijms25073941)
Supplement: Supplementary file 1 [file ijms-25-03941-s001.zip › Captions.pdf]

Supplemental Information

Supplemental Table S1. Detail information of 107 plant genomes.

Supplemental Table S2. Statistics of the *ERF* subfamily genes identified in different plant lineages.

Supplemental Table S3. List of all the *ERF* subfamily genes identified in the 107 angiosperms.

Supplemental Table S4. Node list and edge list of the communities at k =3.

Supplemental Table S5. List of all synteny network community.

Supplemental Table S6. Statistics of the *ERF* tandem genes identified in different plant lineages.

Supplemental Table S7. Detailed information on putative *ERF* tandem genes.

Supplemental Figure S1. Close-up view of the community that generated tandem duplication events, where node shapes represent different communities, node colors represent different lineages, and nodes labeled with "T" represent tandem duplication gene.
